# A systematic review and overview of health economic evaluations of emergency laparotomy

**DOI:** 10.1186/s13741-017-0078-z

**Published:** 2017-11-25

**Authors:** Sohail Bampoe, Peter M. Odor, S. Ramani Moonesinghe, Matthew Dickinson

**Affiliations:** 1 Centre for Anaesthesia and Perioperative Medicine, University College London, Gower St, Bloomsbury, London, WC1E 6BT UK; 2 University College Hospital, 235 Euston Road, London, N1 2BU UK; 3 Royal Surrey County Hospital, Egerton Road, Guildford, GU2 7XX UK

**Keywords:** Emergency laparotomy, Economic evaluation, Healthcare costs

## Abstract

**Background:**

Little is known about the economic impact of emergency laparotomy (EL) surgery in healthcare systems around the world. The aim of this systematic review is to describe the primary resource utilisation, healthcare economic and societal costs of EL in adults in different countries.

**Methods:**

MEDLINE, EMBASE, ISI Web of Knowledge, Cochrane Central Register Controlled Trials, Cochrane Database of Systematic Reviews and CINAHL were searched for full and partial economic analyses of EL published between 1 January 1991 and 31 December 2015. Quality of studies was assessed using the Consensus on Health Economic Criteria (CHEC) checklist.

**Results:**

Sixteen studies were included from a range of countries. One study was a full economic analysis. Fifteen studies were partial economic evaluations. These studies revealed that emergency abdominal surgery is expensive compared to similar elective surgery when comparing primary resource utilisation costs, with an important societal impact. Most contemporaneous studies indicate that in-hospital costs for EL are in excess of US$10,000 per patient episode, rising substantially when societal costs are considered.

**Discussion:**

EL is a high-risk and costly procedure with a disproportionate financial burden for healthcare providers, relative to national funding provisions and wider societal cost impact. There is substantial heterogeneity in the methodologies and quality of published economic evaluations of EL; therefore, the true economic costs of EL are yet to be fully defined. Future research should focus on developing strategies to embed health economic evaluations within national programmes aiming to improve EL care, including developing the required measures and infrastructure.

**Conclusions:**

Emergency laparotomy is expensive, with a significant cost burden to healthcare and systems and society worldwide. Novel strategies for reducing this econmic burden should urgently be explored if greater access to this type of surgery is to be pursued as a global health target.

**Trial registration:**

PROSPERO registration no. 42015027210.

## Background

Emergency laparotomy (EL) is a common procedure that is undertaken in many secondary care hospitals around the world on a daily basis. In the UK, over 30,000 (NELA project team 2015) adult patients undergo the procedure annually with an estimated incidence of 1:1100 (Shapter et al. 2012). In low-income countries, the World Health Organization Emergency and Essential Surgical Care Situational Analysis Tool (SAT) database reveals that 58% of ‘first-level’ facilities currently perform the procedure (Meara et al. 2016). In 2015, The Lancet Commission set a target that by 2030, 80% of the global population should have access to facilities able to safely provide EL within 2 h (Meara et al. 2016). Not only is it a common procedure, it is also associated with substantial mortality, reported variably between 11 and 15% (NELA project team 2015; Shapter et al. 2012). Patients undergoing EL suffer significant morbidity (Howes et al. 2015) with up to 25% still remaining in hospital 20 days after surgery (NELA project team 2015).

Patients may require EL for a multitude of underlying pathologies including malignancy, inflammatory bowel disease, or complications related to previous surgery such as adhesional bowel obstruction. Patients often present with complex multi-morbidity (NELA project team 2015; Howes et al. 2015) which may necessitate higher levels of perioperative care, with many requiring critical care admission. The broad range of potential underlying pathologies and clinical presentations will determine the decision to operate, with conservative, non-surgical management available as an option for some patients. Surgery is, however, often required. According to the International Classification of Diseases – 10 (ICD 10), the total number of procedures that can be included in the coding for EL exceeds 400, reflecting the multitude of presentations and underlying causes (NELA project team 2015; Peden 2011).

In recent years, organisations such as the Department of Health, the National Confidential Enquiry into Patient Outcome and Death and the Royal College of Surgeons of England have expressed particular concerns about the apparent excessive morbidity and mortality that these patients suffer. In the UK, the National Emergency Laparotomy Audit (NELA) has focused interest on the clinical outcomes associated with patients undergoing EL and, alongside other recent studies (NELA project team 2015; Shapter et al. 2012; Howes et al. 2015), confirms that rates of adverse outcomes in these patients are much higher than found in elective surgical patients. However, despite the ubiquity of the procedure and the current drive in the UK to improve the quality of care these patients receive, there is little known about the health economic burden associated with the care of these patients.

The aim of this systematic review is to describe primary resource utilisation, economic and societal costs associated with EL by evaluating and summarising studies undertaking health economic evaluations of this type of major emergency surgery.

## Methods

This study is reported according to PRISMA guidelines (Moher et al. 2009).

### Literature search and selection criteria

The electronic databases MEDLINE, EMBASE, ISI Web of Knowledge, Cochrane Central Register Controlled Trials, Cochrane Database of Systematic Reviews and CINAHL were searched for relevant articles published over a 25-year period between the 1 January 1991 and 31 December 2015. Studies published before this period were not included because we deemed the temporal economic context to be non-comparable with more recent studies. Also included was a search of the ‘grey literature’. For full details of the search strategy, refer to the Appendix.

Eligible studies were full economic evaluation studies, including cost-benefit analyses (CBA), cost-effectiveness analyses (CEA) and cost-utility analyses (CUA). Also included were partial economic evaluations, such as cost description studies and cost analyses, together with randomised trials reporting direct costs or estimates. Our inclusion and exclusion criteria matched those in the National Emergency Laparotomy Audit (NELA project team 2015) in England and Wales; thus, we excluded elective laparotomy/laparoscopy, diagnostic laparotomy/laparoscopy where no therapeutic procedure was performed, appendicectomy, cholecystectomy, non-elective hernia repair without bowel resection; vascular surgery, obstetric surgery and gynaecological surgery. Conference abstracts were excluded.

### Quality assessment

The methodological quality of included studies was assessed using the Consensus on Health Economic Criteria (CHEC) checklist (Evers et al. 2005). This quality assessment tool is specifically designed for systematic reviews of full economic evaluations and assigns a single point for methodological quality as assessed against criteria in each of 19 categories, with a maximum attainable score of 19. The methodological quality of partial economic evaluations was assessed using CHEC checklist items which were applicable (Higgins and Green 2008). Where insufficient detail was reported in the article in relation to a specific category being assessed, no point was awarded for that category. This was applied to both full and partial economic evaluations included in this study.

## Results

**Fig. 1:**
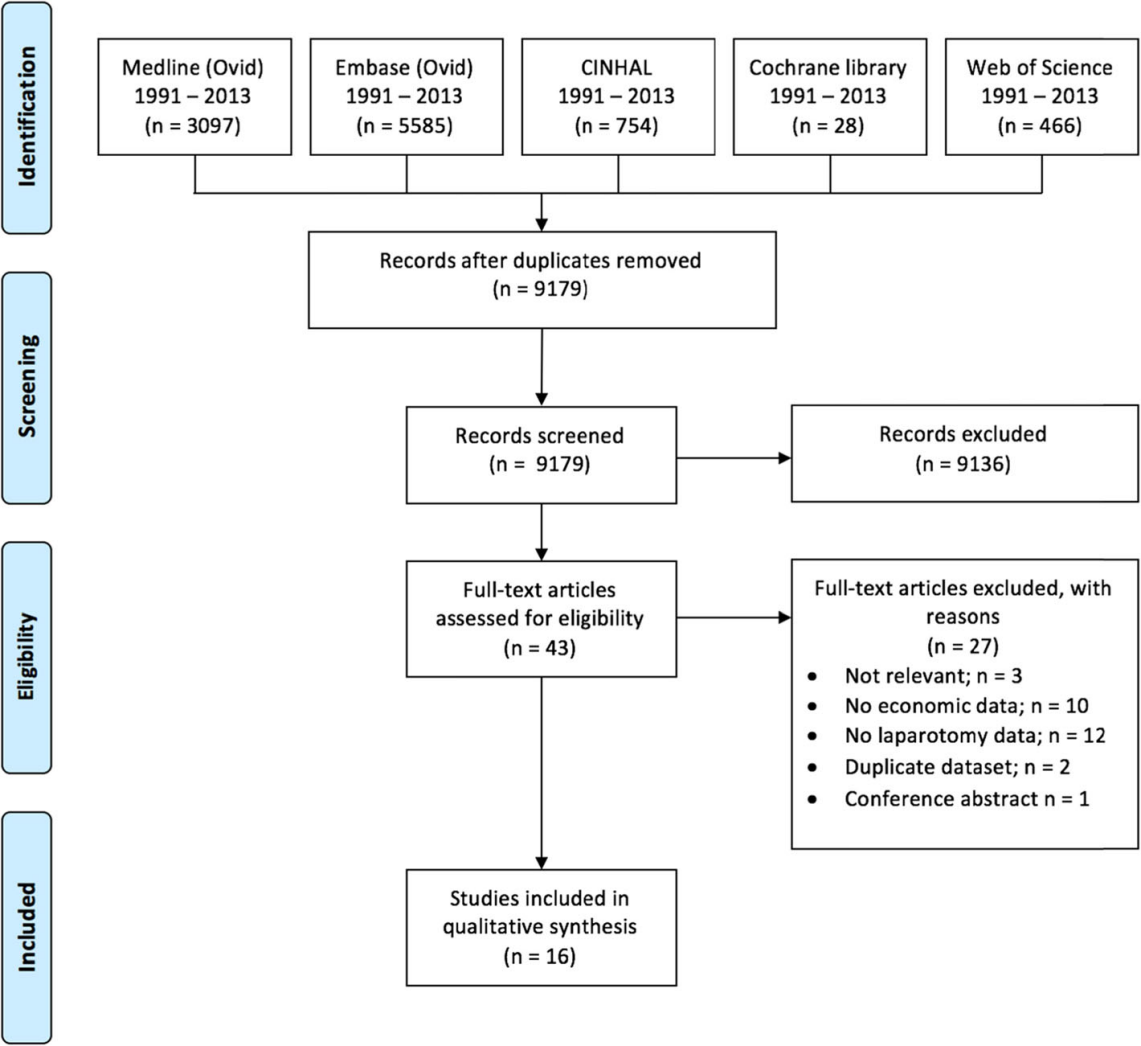
PRISMA flow chart of search process and study selection

The search returned 9179 studies after the removal of duplicates. Titles and abstracts were screened for relevance, and 43 full-text articles that met the inclusion criteria were retrieved. Twenty-seven of these studies were excluded after full-manuscript review by two authors (SB and PO). The remaining 16 studies were included for data extraction. Figure 1 shows the PRISMA flowchart summarising the search and inclusion/exclusion process. Detailed characteristics of the included studies are available in Table 1.

**Table 1:** Abstracted data from eligible studies

Author, Year	Context	Year of data collection	Methodology	Sample size	Outcome measures	Costs calculated by	Length of stay; days	Total cost per inpatient episode	Total cost per theatre episode	ICU costs	Ward costs	Annual national costs	CHEC-list (max. 19)
UK													
Shapter et al. 2012	UK 1 teaching hospital	2009–2010	Partial economic evaluation. Retrospective database analysis using HES codes for emergency laparotomy. Demographic process and outcome data for 2 years in Brighton.	768 (850 ELs)	Inpatient and 30-day mortalities (14 and 11%, respectively) Cost of hospital stay	LOS, HRG codes, HES data extraction	13 (8–24) Median (IQR)	Cost per patient: median (IQR) €8434 (5700–15,103)	€2880 (2200–3840) Median (IQR) £16 per min theatre, 24% of total costs	€6910 (4146–15,202) Median (IQR) £1382 per day ICU, 30% of total costs	€3984 (2256–7050) Median (IQR) £282 per day ward bed, 46% of total costs	£650 m (England)	9
Murray et al. 2012	UK 35 NHS Hospitals	2012	Partial economic evaluation. Prospective national audit of emergency laparotomy patients.	1853	Cost of hospital stay, unadjusted in-hospital mortality	As per Shapter et al. 2012.	15 (9–27). Median (IQR)	Cost per patient: (median (IQR)) £9282 (£6222–£14,400). Cheapest 9 hospitals had a median cost of £7223 (£5418–£11,340); most expensive 9 hospitals were £11,904 (£8224–£17,192)	N/A	N/A	N/A	N/A	5
Menzies et al. 2001	UK 2 District general hospitals	1996–1997	Partial economic evaluation. Retrospective case review of emergency laparotomy for adhesions.	110 (41 treated surgically)	Cost of hospital stay, length of hospital/ICU stay, inpatient mortality	Costs calculated from financial department of each hospital	ICU 0.3 (0.8) Ward 16.3 (11) Mean (SD)	Cost per patient: (mean) £4677.41	£832 per episode, £8.67/min	£386 per day, mean per admission £1332	£180 per day, mean per admission £2941.2	N/A	9
Wilson et al. 1998	UK 1 University hospital	1990–1996	Partial economic evaluation. Retrospective case review of patients with acute bowel obstruction.	26	Direct hospital costs	Unclear how costs derived.	11 (range 2–47) for operative cases	£1964.83 median (no IQR provided)	£470.92 per episode Mean	N/A	£13,581 per 100 patients	N/A	5
US and Canada													
Haider et al. 2015	USA	2001–2010	Partial economic evaluation. Retrospective database analysis. Included patient with elective and emergency surgery for AAA repair, CABG, colon resection for neoplasm. Nationwide Inpatient Sample (NIS) data used. 20% stratified sample, weighted to represent 95% of USA population. Multivariable logistic regression used to investigate the adjusted odds of mortality for elective and emergency cases.	621,925	Cost of hospital stay, length of stay, in hospital mortality	Costs calculated by multiplying total charge by hospital-specific all-payer cost-to-charge ratios. Healthcare cost and utilisation project-NIS discharge-level weights were applied to derive national patient estimates.	Significantly lower mean hospital length of stay for elective patients. All patients= 6.9 ± 5.8 vs. 10.6 ± 7.9 AAA repair = 5.2 ± 6.8 vs 11.1 ± 11.9 CABG = 7.4 ± 5.6 vs 10.3 ± 7.4 colon resection = 6.9 ± 5.2 vs 11.9 ± 7.8	Cost per patient: (mean) US$ 22,616.33 Cost difference for emergency vs. elective care was $8741.22 (30% increase) for AAA repair, $5309.78 (17% increase) for coronary artery bypass graft, and $7813.53 (53% increase) for colon resection.	N/A	N/A	N/A	if 10% of the weighted estimates of patients who underwent emergency procedures had instead been performed electively, the associated cost benefits were $996,169,160 (95% CI, 985,505,565–1,006,834,104)	10
Anantha et al. 2014	Canada 1 Hospital	2009–2010	Partial economic evaluation. Retrospective case-control. Pre- and post- analysis of emergency surgery patients following introduction of an Acute Care and Emergency Surgery Service (ACCESS)	366	Volume of emergency surgery and economic viability of service provision	Surgical billing costs	N/A	Cost per patient (“average”): Pre-pathway = US$ 767.94 Post-pathway = US$ 620.03	N/A	N/A	N/A	N/A	9
Ray et al. 1998	USA National Hospital Discharge Survey database	1994	Partial economic evaluation. Retrospective database analysis of patients with adhesional intestinal obstruction. Hospitalisation costs estimated from 1994 medical provider analysis and review of inpatient expenditure, based on Medicare rates	303,836	Estimation of total number of adhesiolysis admissions in 1994.	Cost calculated according to Medicare records, procedure, diagnosis codes. Surgeon costs calculated from part B Medical Annual Data (BMAD) beneficiary file.	9.7	Daily cost per operative patient: (mean) $1266 per day	$837 per episode Mean	N/A	Admission costs: $1266 per day Mean	$1.3 billion in hospitalisation and surgeon expenditures. $1.1 billion attributed to digestive tract procedures.	7
Khaikin et al. 2007	USA Teaching hospital	1999–2005 (Economic data: 2002–2005)	Partial economic evaluation. Retrospective case review of patients with small bowel obstruction. Comparison of clinical and cost outcomes for laparoscopic vs. open treatment of small bowel obstruction.	62 (31 laparotomy)	Cost of hospital stay, in hospital morbidity (pulmonary, cardiac complications etc.)	Very limited detail. Financial records system used.	Laparotomy = 13 (45% morbidity) Laparoscopic = 7 days (16% morbidity) Mean	Total costs (NB: not per patient): (mean) Laparotomy = $61,855.68 Laparoscopic = $39,866.87	Laparotomy = $11,819.92 Laparoscopic = $9972.07 Mean	N/A	N/A	N/A	1
Europe													
Opmeer et al. 2010	Netherlands 2 Academic and 5 regional teaching hospitals	2001–2005	Full economic evaluation and cost minimisation analysis. Randomised controlled trial (re-laparotomy on demand vs. scheduled). Included patients with emergency laparotomy, severe peritonitis. Compare patient outcomes, health care utilisation and societal cost over 1 year of on-demand vs. planned re-laparotomy).	229 (114 on demand; 115 planned)	Cost of hospital stay, cost after discharge to 1 year after index operation (i.e. societal costs)	Financial records for resource utilisation. Direct and indirect (loss of productivity) costs included.	On demand = 38 Planned = 45 Mean	Cost per patient (mean) On demand group = €65,768 Planned group = €83,450 Mean absolute difference of €17,682 [95%CI €5602–€29,004])	N/A	Total ICU costs (NB, not per day): On demand = €21,040 Planned = €31,248 Mean	Total ward costs (NB, not per day): On demand = €11,609 Planned = €11,748 Mean	Extrapolated savings of €10 million by using an on demand approach (21% reduction in costs)	17
Kössi et al. 2004	Finland 1 university and 4 regional hospitals	1999	Partial economic evaluation. Retrospective case review of patients with adhesional intestinal obstruction. Assessment of surgical workload and direct costs of inpatient care relating to cases caused by previous colorectal surgery.	123 (40 treated surgically)	Cost of hospital stay, length of stay	Cost calculated from hospital specific expenses reported by financial departments Converted to US$ at 1999 rate	7 (SEM 0.6)	Unable to extract data on total cost for emergency laparotomy patients only.	Division of adhesions = $557.50 Adhesiolysis and bowel resection = $1613.50 Mean	N/A	N/A	N/A	8
Tingstedt et al. 2007	Sweden 1 University hospital	1987–1992	Partial economic evaluation. Retrospective case review of patients with adhesional intestinal obstruction, undergoing surgery. Total cost reported are for treating intra-abdominal adhesions—not specified whether patients received emergency laparotomy.	102	Cost of hospital stay, length of stay, societal cost of sick leave	Cost calculated, but source of data not referenced. Sick-leave days retrieved from medical records. Sick leave costs obtained from National Social Insurance Office	N/A Total duration of hospital days over 10 year follow up: 14 (4–163) Median (IQR)	Cost per patient: (mean) €6702 Other costs: Outpatient episode cost = €180 per patient Loss of production cost = total of €366,782	N/A	€1152.29 Mean	N/A	€39.9–59.5 million	7
Rossi et al. 2006	Italy 51 Hospitals	1999–2000	Partial economic evaluation. Retrospective case review of patients with adhesional intestinal obstruction, undergoing “Major abdominal surgery”. Does not explicitly define as emergency laparotomy.	1034 (28 unscheduled major abdominal surgery patients)	Cost of ICU stay, length of ICU stay	Cost calculated from ICU-specific expenses reported as prices paid or national costs for investigational procedures	N/A	N/A	N/A	Total ICU cost (NB, not per day): €3529 (3854) Mean (SD) Mean cost was comprised of variable costs associated with drugs (21.7%), nutrition (4.5%), infusions including blood and blood products (41.6%) consumables (6.4%), imaging (3.9%), laboratory tests (20.2%) and physician consultations (1.5%)	N/A	N/A	7
Kössi et al. 2003	Finland 1 university and 4 regional hospitals	1999	Partial economic evaluation. Retrospective case review of patients with adhesional intestinal obstruction. Assessment of surgical workload and direct costs of inpatient care.	138 (40 treated surgically)	Annual direct hospital costs	Cost calculated from hospital specific expenses reported by financial departments Converted to £ at 1999 rate	11 (2–34) for surgical patients Median (IQR)	Annual total direct hospital costs (NB: not per patient): £181,653	N/A	N/A	N/A	£2.1 million	8
Ivarsson et al. 1997	Sweden 1 University hospital	1997	Partial economic evaluation. Prospective observational study of patients with adhesional bowel obstruction.	57	Direct hospital costs	Costs calculated from financials records of hospital. Rehabilitation costs based upon council charges and time required. Sick leave charges.	N/A	Cost per patient (mean): Medical care: US$4999.03 Total expenditure: US$5694.12	N/A	N/A	N/A	Extrapolation of costs to Sweden from admission estimates: $13 million pa	6
Rest of the world													
Alwan et al. 1999	New Zealand 1 Teaching hospital	1988–1996 Only cost analysed for cases between 1993 and 1996	Partial economic evaluation. Retrospective case review of patients with adhesional intestinal obstruction.	332 (253 treated surgically)	Resource implications of managing small bowel obstruction	Costs calculated by LOS × daily hotel costs, investigation costs, support services, medical staff anaesthesia and operating theatre costs. Adjusted to 1996.	10 (4–18) Median (IQR)	Cost per patient (mean) NZ$7630 (2038–135,173) Daily cost per operative patient: NZ$1264 ($803–3741) per day	N/A	N/A	N/A	N/A	8
Koh et al. 2013	Singapore 1 University teaching hospital	2006–2011	Partial economic evaluation. Retrospective case-control review of all patients undergoing emergency laparoscopic colectomies, matched with open colectomies.	46	Cost of hospital stay, length of stay, complications	Financial records for resource utilisation	Laparotomy = 7 (3–31) Laparoscopic = 6 (3–28) Median (IQR)	Cost per patient: Median (IQR) Laparotomy = US$ 11,300 ($5080 -$33,530); Laparoscopic = US$12,360 ($6590–40,920)	Laparotomy = $3500 ($3000–$8580) Laparoscopic = $4050 ($3220–$8420) Median (IQR)	N/A	N/A	N/A	7

Many included studies did not score highly on the CHEC checklist, reflecting our observation that most economic evaluations of EL are direct cost analyses and therefore represent partial economic evaluations rather than full economic evaluations. The highest scoring paper was Opmeer et al. (Opmeer et al. 2010): this was the only study to perform a full economic analysis, including a cost minimisation analysis.

Only 5 of the 16 studies measured health economic outcomes of only EL surgery as the primary outcome, with the remainder including EL surgery as a subgroup of within a larger cohort being investigated. The degree of heterogeneity between studies—in particular, the types of economic evaluation conducted and the range of outcomes reported—was substantial enough to preclude data synthesis. Extracted data from all the included 16 papers are presented in Table1 (Appendix).

### United Kingdom

Four studies with a total of 2757 patients reported on health economic outcomes for EL surgery in the UK, over a period of 22 years from 1990 to 2012. All were partial economic analyses, reporting direct healthcare costs and reported total inpatient episode cost as an outcome measure. All studies except Wilson et al. (Wilson et al. 1998) explicitly declared their methodology for cost calculation, which included analysis of data of patient length of stay and hospital resource utilisation costs.

The highest quality of these studies, both in terms of CHEC score and size, was led by Shapter et al. (2012) (CHEC score = 9). They reported a median inpatient cost in a single UK institution in 2009/10 of €8434 (IQR 5700–15,103) and from this projected that the annual national inpatient cost of EL was approximately £650 million (Shapter et al. 2012).The authors also calculated the actual reimbursement, per patient, received by the hospital, using the centrally allocated payment by results (PbR) HRG codes. This is the system by which healthcare providers receive funding from central government in the UK. They found that the mean income of £6905 received created a loss of approximately £6100 per patient. When extrapolated nationally, this equates to a reimbursement shortfall of approximately £300 million for the NHS (Shapter et al. 2012).

Menzies et al. (CHEC = 9) used ICD-10 codes to retrospectively identify 110 patients with adhesive small bowel obstruction, admitted to two English district general hospitals between 1996 and 1997 (Menzies et al. 2001). Forty-one patients (37%) were treated surgically, and the associated inpatient costs, including referral, diagnostic, admission and follow-up costs, came to a median of £1964.83 (Menzies et al. 2001).

The authors of the first report of the UK Emergency Laparotomy Network (NELA project team 2015) performed a post hoc analysis using the same cost assumptions as Shapter et al. Using this methodology, Murray et al. (CHEC = 5) calculated a median cost per patient of £9282 (IQR £6222–14,400) (Murray et al. 2012).

Finally, Wilson et al. (CHEC = 5) reported a retrospective cross-sectional review of 59 patients presenting with small bowel obstruction in a single UK teaching hospital (Wilson et al. 1998) and who received either surgery or conservative treatment. The authors calculated that the median cost per admission for surgery, with a median length of stay of 11 days, would be £1964.83 (Wilson et al. 1998).

### USA and Canada

Four studies reported on health economic outcomes for major surgery, including EL, in North America, incorporating database records for a total of 926,189 patients and spanning a period of 16 years between 1994 and 2010. All four were partial economic analyses, reporting direct healthcare costs. Two were large retrospective database analyses, reporting estimated per patient costs based upon hospital length of stay and healthcare provider costs. Despite occurring over similar time frames, results from the North American studies demonstrate wide ranges in hospital charges associated with EL surgery.

Haider et al. (Haider et al. 2015) (CHEC = 10) identified 48,599 patients undergoing emergency colonic resection between 2001 to 2010 in the USA, at a mean cost of $22,616.33 per patient—$7813.53 (CI 7746.33–7880.72) more expensive than elective colonic resection in the same population. Healthcare costs in surgical patients with acute intestinal obstruction secondary to adhesions were identified by Ray et al. (Ray et al. 1998) (CHEC = 7). Mean daily costs per operative patients were much lower than found in the Haider study, at $1266/day for a mean length of stay of 9.7 days.

Anantha et al. (CHEC = 9) conducted a single-centre retrospective longitudinal study in which surgical costs for emergency surgery were compared before and after the introduction of a new dedicated emergency general surgery service in Canada (Anantha et al. 2014). Cost per patient decreased significantly from $767.94 to $620.03 following introduction of the program. Finally, Khaikin et al.’ s (CHEC = 1) retrospective review matched 31 patients who underwent a laparotomy with patients who underwent laparoscopic treatment for acute adhesive small bowel obstruction (Khaikin et al. 2007). Mean operative charges and total hospital charges for laparotomy and laparoscopy were US$9972.07 vs. US$11,819.92, respectively. The authors hypothesised that the higher cost of laparoscopic surgery was due to longer operating times and equipment costs (Khaikin et al. 2007).

### Europe

Six studies published between 1992 and 2005 and including 1683 patients reported on health economic outcomes in European countries (excluding the UK).

Opmeer et al. (Higgins and Green 2008) (CHEC = 17) undertook a full economic evaluation comparing patient outcome, health care utilisation and costs of on-demand and planned re-laparotomy following initial laparotomy in patients with severe peritonitis in seven Dutch hospitals between 2001 and 2005 (Opmeer et al. 2010). The authors used a cost minimisation analysis to determine economic differences. At 12 months follow-up, including the index admission, the mean direct medical costs per patient for the on demand group were calculated at €62,742 (US$86077) compared to €81,532 (US$111858) for the planned re-laparotomy group. A societal perspective cost minimisation analysis was also performed and included funds generated from direct medical costs, direct non-medical costs, e.g. travel to and from healthcare providers, and indirect costs, e.g. loss of productivity due to inability to work. The societal cost per patient associated with re-laparotomy was €4617 (on demand) vs. €6641 (planned); mean costs per patient generated by the ICU stay (€21,040 for the on demand group vs. €31,248 for the planned group), mean in-hospital and 12-month follow-up direct medical costs per patient were €14,418 in the planned re-laparotomy group and €4069 lower in the on demand group (Opmeer et al. 2010).

The remaining five studies from Europe were partial economic evaluations reporting direct healthcare costs. Rossi et al. (CHEC = 7) were the only group to exclusively study ICU costs in a prospective, observational study of 51 ICUs in Italy (Rossi et al. 2006). The mean variable ICU cost per patient undergoing unscheduled abdominal surgery was €3529 (SD €3854) (Rossi et al. 2006). The remaining four European studies primarily looked at cost associated with adhesional bowel obstruction with surgical costs reported as sub-analyses.

In 1997, Ivarsson et al. (CHEC = 6) undertook a small prospective study and reported the direct costs associated with bowel obstruction resulting from adhesions that required surgery. The authors estimated that in Sweden, this condition might cause 2330 hospital admissions per annum, equating to an estimated cost of US$13 million (Ivarsson et al. 1997).

Tingstedt et al. (CHEC = 7) performed a retrospective cost analysis calculating the total cost of adhesion-related problems for 102 patients following bowel surgery between 1987 and 1992 that included operative and non-operative treatment. The mean cost of treating patients with postoperative adhesions was calculated as €6702 per inpatient admission. The authors extrapolated this figure to include outpatient visits and readmissions to achieve a figure of €806,940 (Tingstedt et al. 2007). They also included costs calculated for sick leave and loss of productivity, based on the Swedish National Social Insurance Office figures, producing a figure of €1,198,771 annually for the 270,000 people living in the catchment area of the hospital. They then further extrapolated this amount to take in to account the total population of Sweden, estimating an annual cost of between €39.9 million and €59.5 million, depending on the accuracy of the clinical coding (Tingstedt et al. 2007).

The two studies authored by Kossi et al. (Kössi et al. 2004; Kössi et al. 2003) collected information about surgical workload and the direct costs of inpatient care of patients admitted with intestinal obstruction in five hospitals in Finland in 1999. In their first manuscript, (CHEC = 8), the authors calculated that annual direct hospital costs were £181,653 and extrapolated that to a sum of £2,077,796 per annum for the whole of Finland (Kössi et al. 2004). Their later analysis (CHEC = 7) analysed 123 admissions during which 40 patients required 176 operations. A sub analysis of those patients who had surgery due to adhesions secondary to colorectal surgery calculated mean inpatient costs to be $1613.50.

### Singapore and New Zealand

Koh et al. performed a case-matched retrospective review of patients who had undergone either emergency laparoscopic or open colectomies, with 23 patients in each group. There was no significant difference between the groups for severities and types of perioperative complications or length of stay (Koh et al. 2013). They included procedural (e.g. operating room charges), nonprocedural (e.g. laboratory and radiological investigations, medications and consumables) and therapy costs (e.g. physiotherapy). Median total costs were US$11300 vs. US$12360 in the open and laparoscopic groups, respectively (Koh et al. 2013).

Alwan et al. conducted a retrospective review of all patients admitted as an emergency that had a diagnosis of small bowel obstruction recorded, in a New Zealand teaching hospital between 1988 and 1996. There were 374 hospital admissions, with 68 patients (20.5%) developing a total of 102 complications and a mortality rate of 2.4% (eight patients) (Alwan et al. 1999). They included use of hotel, investigations, support services, medical staff, anaesthesia and the use of the operating theatre in their costs. The costs were adjusted to 1996 rates, giving a mean daily cost of NZ$1264 (range NZ$803–3741) and an overall cost of NZ$7630 (range NZ$2038–135,173) for patients who underwent an operative procedure (Alwan et al. 1999).

## Discussion

This review identified a wide range of international studies describing the direct hospital and societal costs associated with EL over a period of almost 20 years. Our results demonstrate heterogeneity in the methodological quality of economic evaluations of EL surgery, demonstrating a need to improve study design in order to more accurately inform decisions on resource allocation. This heterogeneity precludes meta-analysis of existing research findings. Each evaluation must be considered within the context of the local health system in which the study was performed and relevant time horizon. Bearing these limitations in mind, the most contemporaneous studies indicate that in-hospital costs for EL are in excess of US$10,000 per patient episode, rising substantially when societal costs are considered.

### Quality and methodological limitations

The majority of studies report the direct resource utilisation costs associated with emergency abdominal surgery and as such are classified as partial economic evaluations. Most were relatively poor quality economic evaluations, as assessed using the CHEC list (Evers et al. 2005). The studies ubiquitously used different methods for the calculation of costs. Most relied on retrospective analysis of various national databases or financial databases associated with their institutions. Calculations of cost varied between studies based on the variable identification of components of care associated with EL surgery.

Costs were also reported at different levels of context within different healthcare systems. Two out of three American studies (Haider et al. 2015; Ray et al. 1998) reported costs at national level, perhaps reflecting easier access to national patient databases. These manuscripts reported estimates of national costs in the region of a billion US dollars. Three European (Opmeer et al. 2010; Ivarsson et al. 1997; Tingstedt et al. 2007) and one British reports (Shapter et al. 2012) also presented estimates of national cost. These costs varied between tens and hundreds of millions suggesting that not only is the treatment of these patients expensive, but also that there is a huge variation in the amount that this care costs between nations. Only two studies, both European, report costs from a societal perspective including costs of sickness and lost income (Opmeer et al. 2010; Tingstedt et al. 2007).

### Key findings

Importantly, Shapter et al. estimated a £300 million shortfall in reimbursement funding for EL from a national perspective in the UK (Shapter et al. 2012). This may suggest a disparity between the perceived costs of EL when compared to the actual measured costs. This disparity may occur because of the observed variation in morbidity and hospital length-of-stay that occurs with all surgery, but especially emergency surgery. Reimbursement is generally in the form of bundle payments based on the estimated average cost. However, the actual average cost may be higher than the estimate due to long-staying outliers. This potential disparity is an important consideration when planning hospital services, particularly in the context of The Lancet Commission’s aim to increase access to facilities that can perform EL to 80% of the population worldwide (Meara et al. 2016). Implementing this may have significant cost implications for many health economies around the globe that may already be fragile. An accurate prediction of the costs associated with achieving this aim within each health system is therefore vital to prevent any financial shortfall.

It is possible that many of the studies in this review have underestimated the true costs associated with EL. Only two studies (Opmeer et al. 2010; Tingstedt et al. 2007) reported the societal costs associated with EL, for example, the costs associated with loss of productivity and sick leave costs. This may reflect a difficulty in measuring these outcomes in emergency patients, compared to the elective population. There are likely to be societal costs which are difficult to measure such as the costs of rehabilitating elderly patients after EL and the costs associated with the long-term care of those who cannot be rehabilitated back to full independence. Almost half of all EL patients audited by NELA (NELA project team 2015) in the UK were over 70 years of age, meaning that societal costs from loss of working income may not be as great as for surgical pathologies affecting those of a younger demographic. Metrics that can be used to assess societal costs, such as Health Related Quality of Life (HRQOL) surveys, can be analysed to provide Quality Adjusted Life Years; not one study we identified used such measures. This may be because HRQOL instruments require both baseline measurements (i.e. pre-operative) and subsequent follow-up measurement; baseline measurements in particular may be difficult to obtain in patients who are critically unwell with an acute abdominal pathology. There is therefore a case for research which investigates the validity of surrogate or retrospective assessment of baseline function, which might be more appropriately used in the emergency surgical setting.

Only five studies (Shapter et al. 2012; Opmeer et al. 2010; Menzies et al. 2001; Rossi et al. 2006; Tingstedt et al. 2007) reported ICU costs. As evidence accumulates that high-risk surgical patients, such as those undergoing EL, may benefit from early and routine post-operative critical care admission (NELA project team 2015; NCEPOD—POC 2011; Emergency Surgery Standards for unscheduled surgical care 2011), it is important to consider that the specific data described in this review suggests that daily ICU cost is almost twice as expensive to healthcare providers as ward-based care (Shapter et al. 2012; Opmeer et al. 2010). This finding has important consequences for financial planning in the setting of emergency surgery service delivery.

There are some limitations to this review. First, we may have missed some older analyses of health economic outcomes as the date range for our search spans a period of only twenty-five years. After careful consideration, we felt that the inclusion of earlier studies would make meaningful economic comparison difficult due to substantial differences in temporal context. The start date was chosen to coincide with the release of the first National Confidential enquiry into Perioperative Deaths (NCEPOD) report, which at the time sparked increased interest in perioperative outcomes after high-risk surgery. Second, a multitude of conditions and procedures can be coded as EL, with some authors suggesting up to 400 different variations (Peden 2011). We therefore used broad procedural terms in order to capture as many relevant studies as possible; however, it is possible that some relevant studies may have been missed.

Finally, the biggest limitation is the quality of the constituent studies themselves, and this provides the justification for our main recommendations. The heterogeneous methodologies of studies identified in this systematic review, coupled to the dynamic nature of the healthcare systems in which they were performed, mean that, at best, each study represents a snapshot into the health economics of EL, relevant to time and location of study conduct. Nevertheless, it is apparent that the context specific, immediate in-hospital and post-discharge requirements of EL patients represent a significant cost implication for healthcare providers and national healthcare funders, particularly when compared to equivalent elective surgical procedures. The literature is also limited by the single-centre nature of many of these studies.

## Conclusions

The literature demonstrates wide variation in quality and outcomes between different healthcare providers even within a single healthcare system (NELA project team 2015; Murray et al. 2012), and therefore, it is likely that there is also substantial variation between institutions in the costs incurred.

National measurement programmes, such as NELA in the UK and the National Surgical Quality Improvement Program (NSQIP) in the USA, provide a unique opportunity for health economic analyses to be undertaken using data capture mechanisms embedded within health services. In particular, the addition of HRQOL measures to the datasets would provide the opportunity for societal impact to be better assessed and for the variation in costs and cost effectiveness between providers to be highlighted. For this to be feasible, research is required into the use of surrogate or retrospective recall of baseline QOL data and the acceptability of this type of measurement to patients undergoing such a high-risk procedure (and their relatives). The widespread adoption of electronic health record systems may also present a further opportunity to routinely capture the data required for cost analyses and comparison of different surgical techniques or perioperative pathways. Consideration of these issues is particularly important given the current focus on improving outcomes for EL patients: this has led to a welcome proliferation of innovative pathways and treatments under consideration in clinical trials (Pearse 2014; Edwards 2017). If any of these interventions demonstrate efficacy in the research setting, then economic analyses undertaken alongside evaluations of implementation and clinical effectiveness will assist health services in planning appropriate resource allocation—an issue which has already been highlighted by work contained within this systematic review as requiring consideration (Shapter et al. 2012). An argument could be made for the centralisation of EL services, restricting significant financial losses to fewer specialist centres with the potential added benefit of also improving the quality of services provided that has been observed with the centralisation of other acute services such as stroke and primary percutaneous intervention following myocardial infarction. This approach may however restrict access to such emergency surgical services for many populations, especially in low- and middle-income countries, and may hinder progress towards the Lancet Commission’s aim of 80% of the global population having access to facilities able to safely provide EL within 2 h.

Alternative solutions for reducing the economic burden of such surgery should be urgently explored. Alternative strategies such as the introduction of enhanced recovery pathways and bundles of evidence-based care, such as the Emergency Laparotomy Pathway Quality Improvement Bundle (ELPQuIC), have been shown to improve outcomes such as mortality (Huddart et al. 2015) and may in time also show a reduction in costs by reducing length of stay and complications after surgery. Future research should focus on evaluating the cost-effectiveness of quality improvement initiatives in EL, consider both hospital and community care, in order to highlight optimal strategies for improving ‘wrap around’ EL care. Whilst reducing inpatient length of stay has a reciprocal effect on community healthcare providers, in the UK, this approach has demonstrated combined savings (Costing statement: Implementing the NICE guideline on Transition between inpatient hospital settings and community or care home settings for adults with social care needs (NG27) 2015).

Caution is required in drawing conclusions from the constituent data in this review because of the variable quality of health economic studies. However, it is clear that EL is a high-cost and common procedure which would benefit from better quality research, including the interrogation of routine data enabling the measurement of cost, and the validation of processes for HRQOL measurement in emergency patients.

## Appendix

### Search strategy

The following key words and Medical Subject Headings (MESH), which included relevant wildcard phrases, were used: “emergency laparotom*”, “acute abdomen”, “emergency abdom* surger*”; and combined, using Boolean operators, with the following health economic key words and MESH terms, and the relevant wildcard phrases: “cost*”, “economic evaluation”, “cost effectiveness analys*”, “CEA”, “cost utility analys*”, “CUA”, “cost benefit analys*”, “CBA”, “health econom*”, “health utilit*”, “quality of life”, “quality adjusted life year*”and “QALY”.

The grey literature search included a search of Google Scholar and the following organisation based websites: Royal College of Surgeons of England, Association of Surgeons of Great Britain & Ireland, Association of Coloproctology of Great Britain & Ireland, Royal College of Anaesthetists, Association of Anaesthetists of Great Britain & Ireland, NHS Networks EL Network, Department of Health, The Health Foundation, NHS Economic Evaluation Database, the World Health Organization, The Rand Corporation, SIGLE—System for Information.

## Abbreviations

AAA: Abdominal aortic aneurysm
CABG: Coronary artery bypass graft
CBA: Cost-benefit analyses
CEA: Cost-effectiveness analyses
CHEC: Consensus on Health Economic Criteria
CUA: Cost-utility analyses
EL: Emergency laparotomy
HES: Hospital episode statistics
HRG: Healthcare resource group
HRQOL: Health Related Quality of Life
ICD 10: International Classification of Diseases – 10
ICU: Intensive care unit
IQR: Inter-quartile range
NELA: National Emergency Laparotomy Audit
NSQIP: National surgical and quality improvement programme
PBR: Payment by results
PRISMA: Preferred Reporting Items for Systematic Reviews and Meta- Analyses
SAT: Situational analysis tool
SD: Standard deviation
